# Supplementary material for: Evolution from the plasmon to exciton state in ligand-protected atomically precise gold nanoparticles
Source: Nat Commun. 2016 Oct 24;7:13240. doi: 10.1038/ncomms13240 (PMC5078994; doi:10.1038/ncomms13240)
Supplement: Supplementary Information — Supplementary Figures 1-9, Supplementary Notes 1-4 and Supplementary References. [file ncomms13240-s1.pdf]

Supplementary Figures

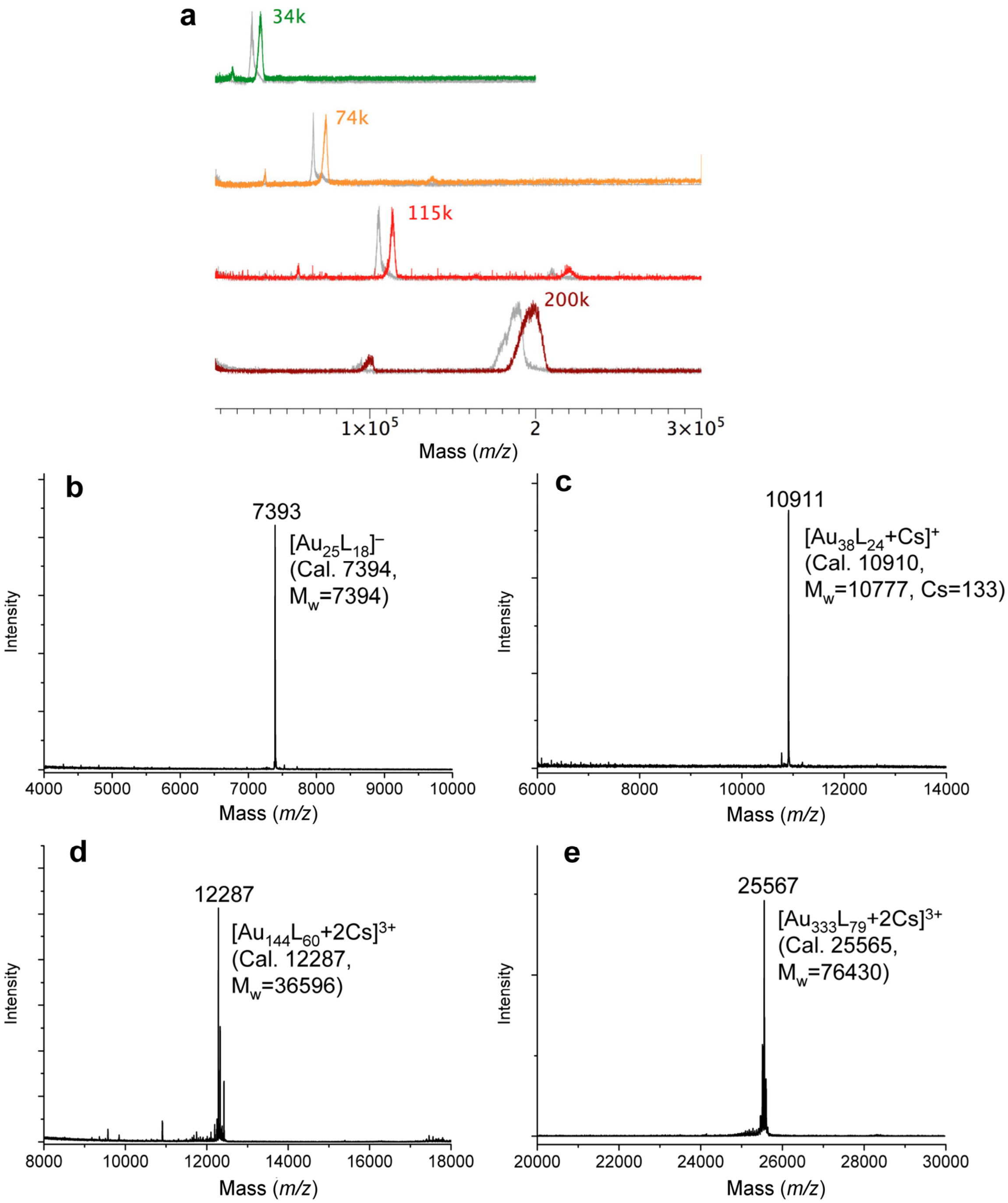

Supplementary Figure 1. Mass spectrometry characterization of Au_{25}, Au_{38}, Au_{144}, Au_{333}, $Au_{\sim 520}$ and $Au_{\sim 940}$ nanoclusters. (a) MALDI-mass spectra of Au_{144}, Au_{333}, $Au_{\sim 520}$ and $Au_{\sim 940}$; the grey profiles are the spectra measured at high laser intensity, and the colored profiles are the spectra measured at low laser intensity. (b) to (e) ESI-mass spectra of Au_{25}, Au_{38}, Au_{144}, and Au_{333} (note: $Au_{\sim 520}$ and $Au_{\sim 940}$ are too large for ESI analysis).

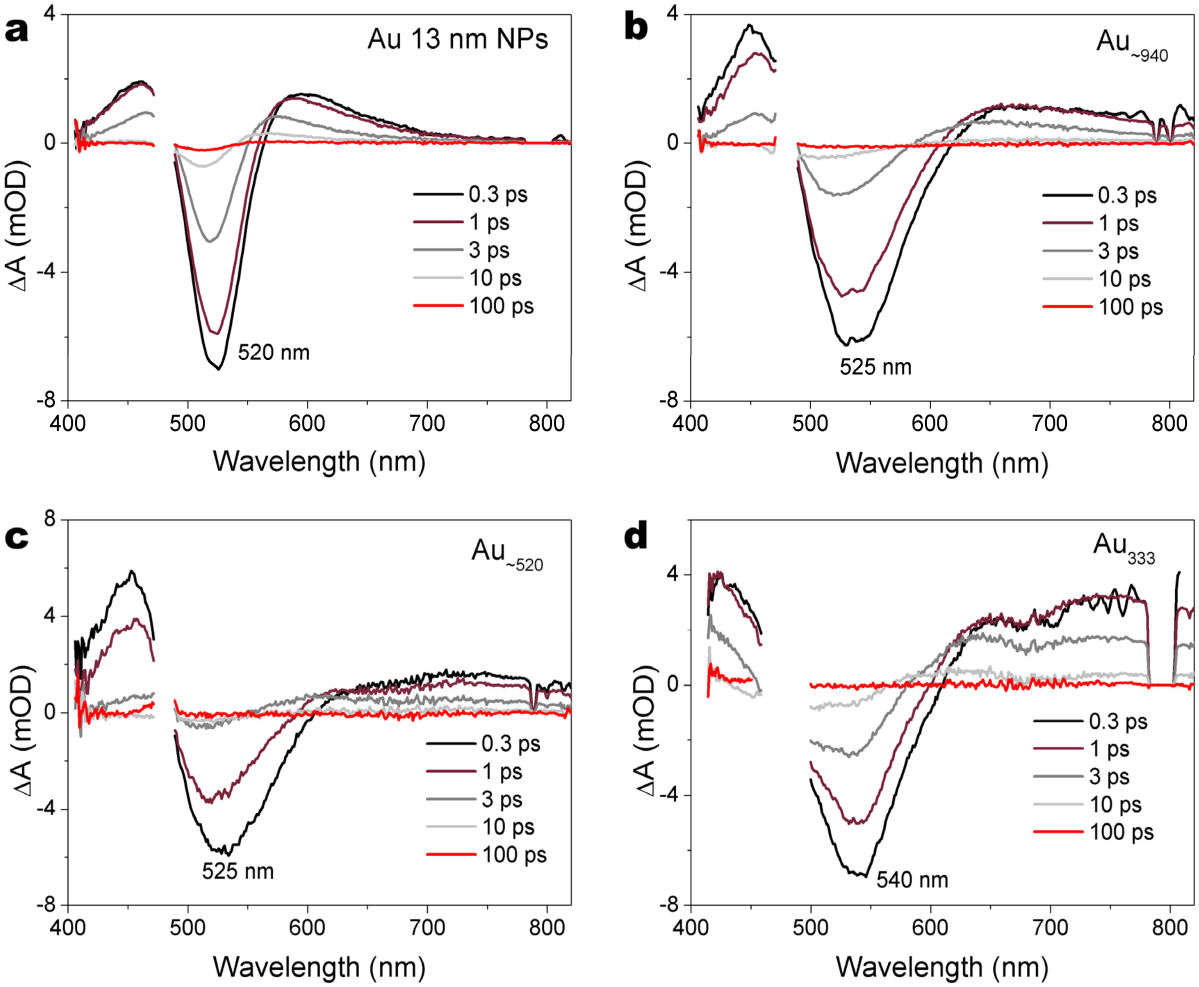

Supplementary Figure 2. Transient absorption spectra as a function of time delay for gold nanoparticles. (a) Au 13 nm NPs, (b) $Au_{\sim 940}$, (c) $Au_{\sim 520}$ and (d) Au_{333} with 480 nm pump. Transient absorption spectra at 0.3 ps, 1 ps, 3 ps, 10 ps, 100 ps were monitored for all gold nanoparticles. Residual scattering of the pump beam around 480 nm were cut off for all samples.

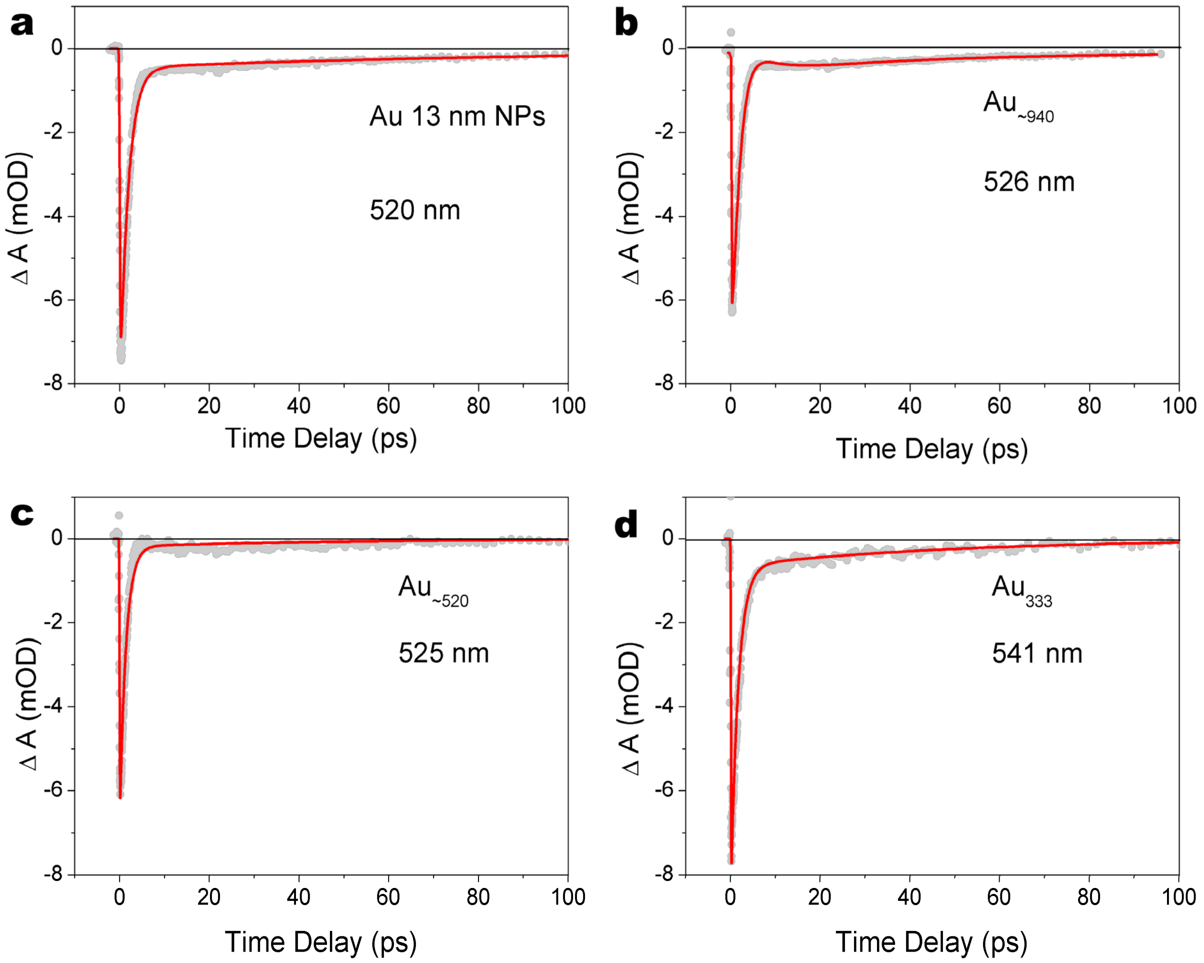

Supplementary Figure 3. Electron dynamics of gold nanoparticles. Kinetic traces (grey dot) and the corresponding fit (red solid line) at the maximum of SPR for (a) Au 13 nm NP, (b) $Au_{\sim 940}$, (c) $Au_{\sim 520}$, (d)Au_{333} with 480 nm pump.

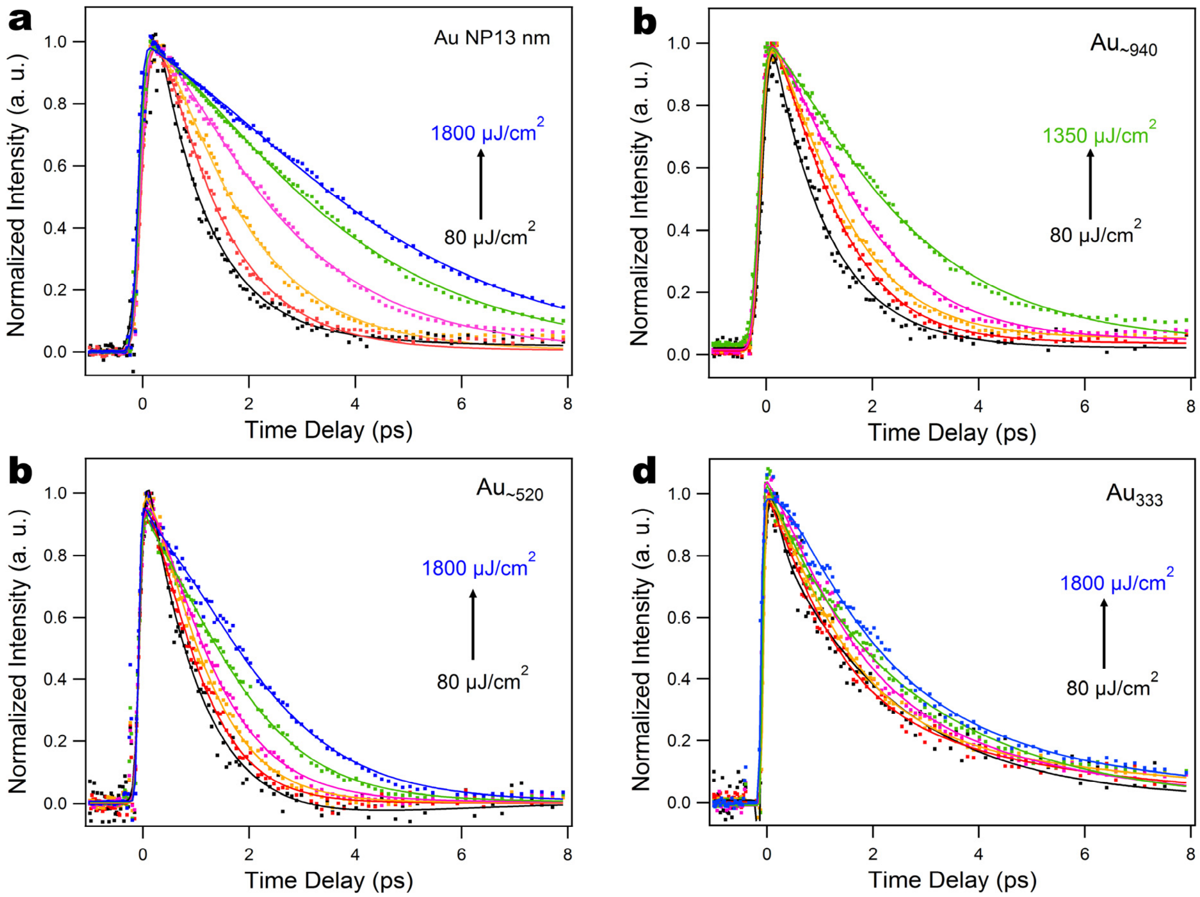

Supplementary Figure 4. Normalized decay kinetics and corresponding fit as a function of pump fluence for plasmonic gold nanoparticles. (A) Au 13 nm nanoparticles, (B) $Au_{\sim940}$, (C) $Au_{\sim520}$, (D)Au_{333}.

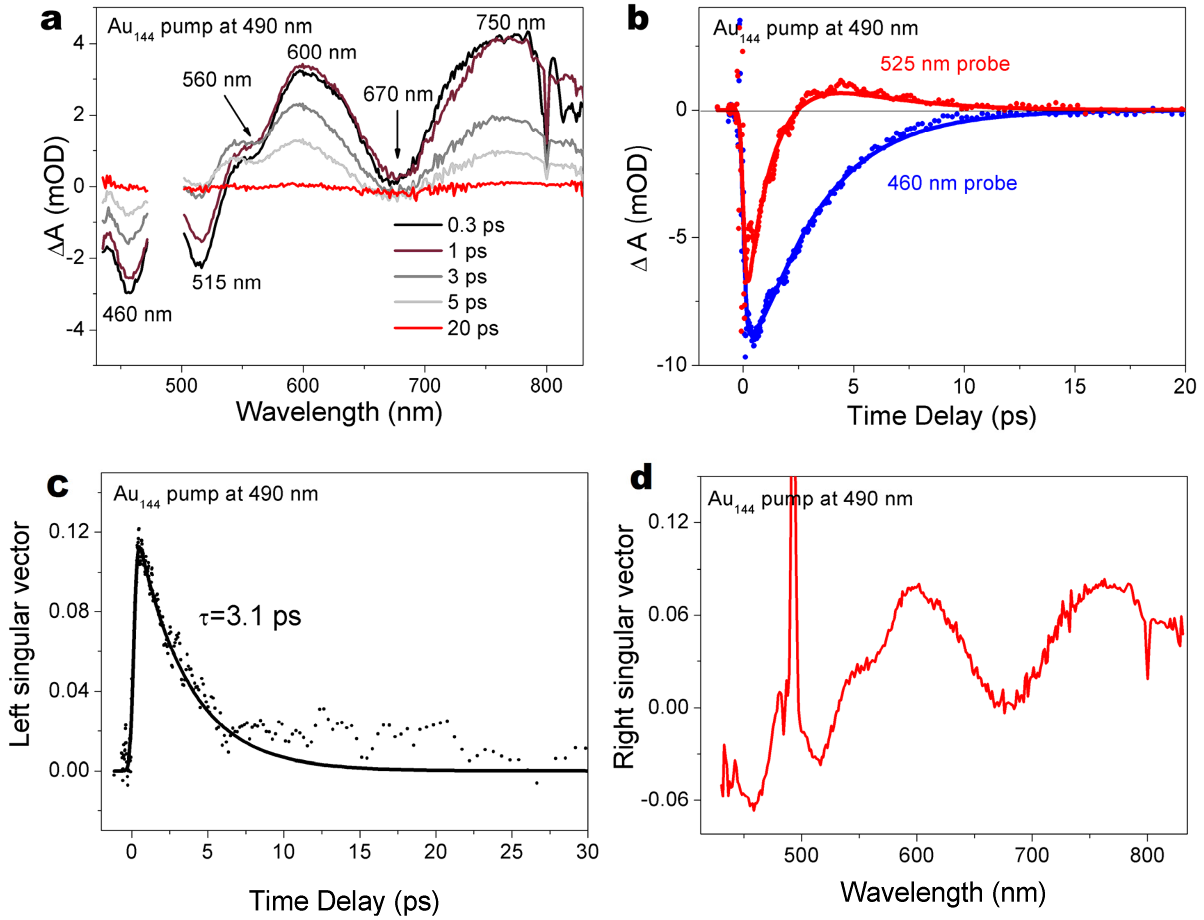

Supplementary Figure 5. Excited state behavior of Au_{144}. (a) Transient absorption spectra as a function of time delay of Au_{144} with 490 nm excitation. (b) Kinetic traces (dot) and fit (solid line) at 460 nm (blue) and 525 nm (red) of Au_{144} with 490 nm excitation. (c) Left singular vector and the corresponding fit obtained from singular vector decompose (SVD). (d) Right singular vector obtained from SVD, the sharp artifact around 490 nm is due to the pump laser. SVD analysis suggests that there is one decay component for Au_{144} with time constant of 3.1 ps.

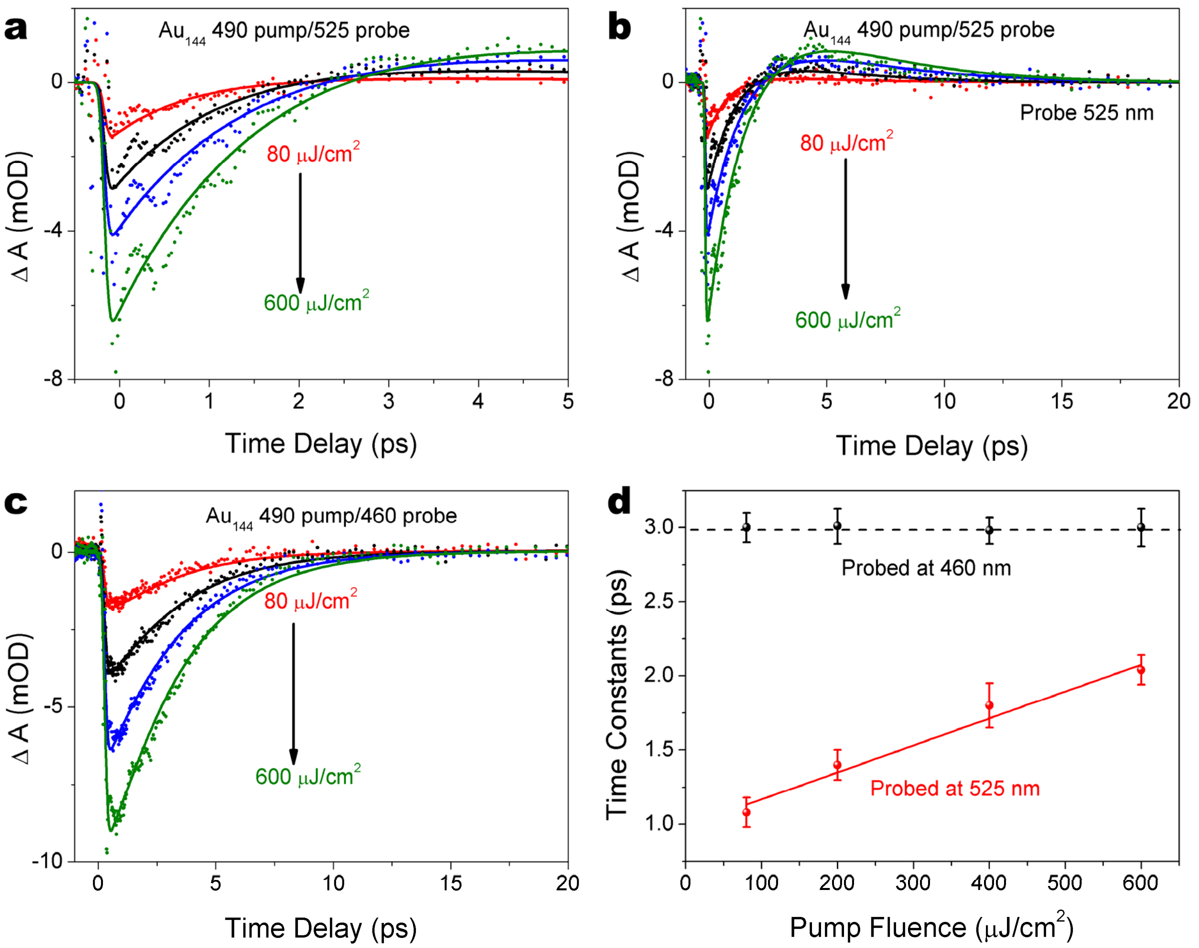

Supplementary Figure 6. Comparison between kinetic traces around 525 nm and 460 nm of Au_{144}. Kinetic traces at 525 nm as a function of pump fluence and the corresponding fit between 0-5 ps (a) and 0-20 ps (b). (c) Kinetic traces at 460 nm as a function of pump fluence and the corresponding fit. (d) The extracted time constants of kinetic traces probed at 460 nm (black) and 525 nm (red). The error bars in (d) represent the standard deviation of multiple measurements under the same conditions.

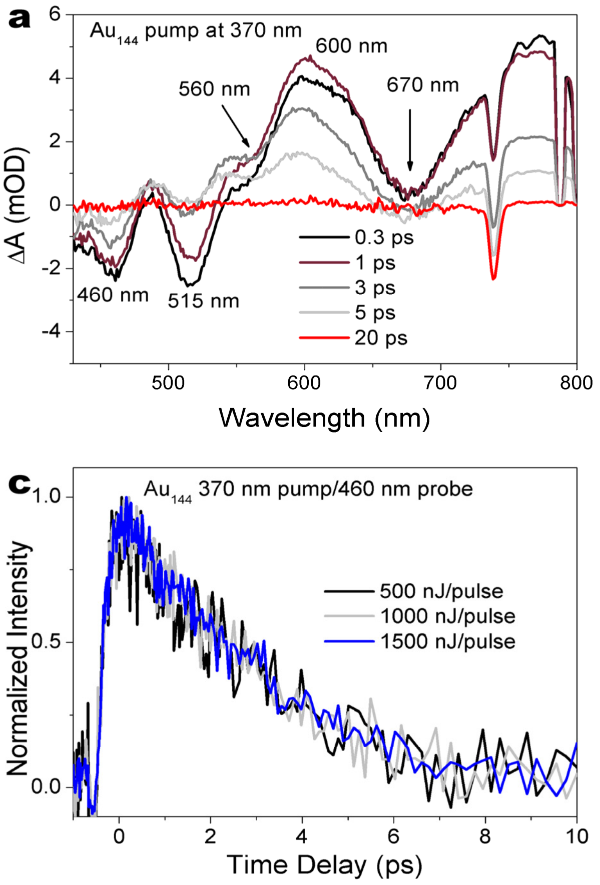

Supplementary Figure 7. Transient absorption measurement on Au_{144} with different excitation wavelength (a) (b) Transient absorption spectra as a function of time delay of Au_{144} with 370 nm and 700 pump. (c) (d) Kinetic traces at 460 nm as a function of pump power of Au_{144} with 370 nm and 700 nm pump. The sharp peak in (a) is due to the residual scattering of the pump pulse.

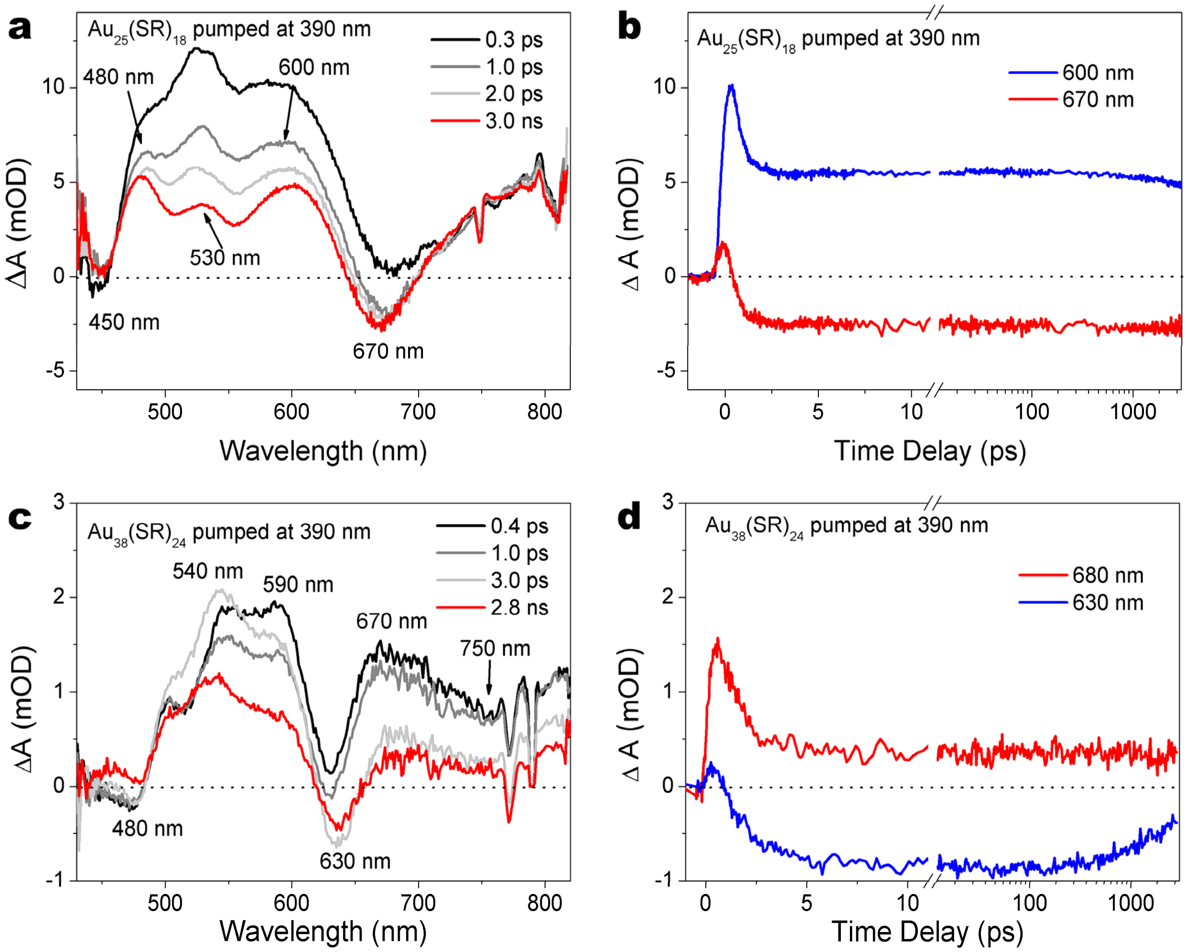

Supplementary Figure 8. Transient absorption spectra and kinetic traces of Au_{38} and Au_{25}. (a) Transient absorption spectra as a function of time delay and (b) selected kinetic traces of $Au_{25}(SR)_{18}$ with 390 nm excitation. (c) Transient absorption spectra as a function of time delay and (d) selected kinetic traces of $Au_{38}(SR)_{24}$ with 390 nm excitation.

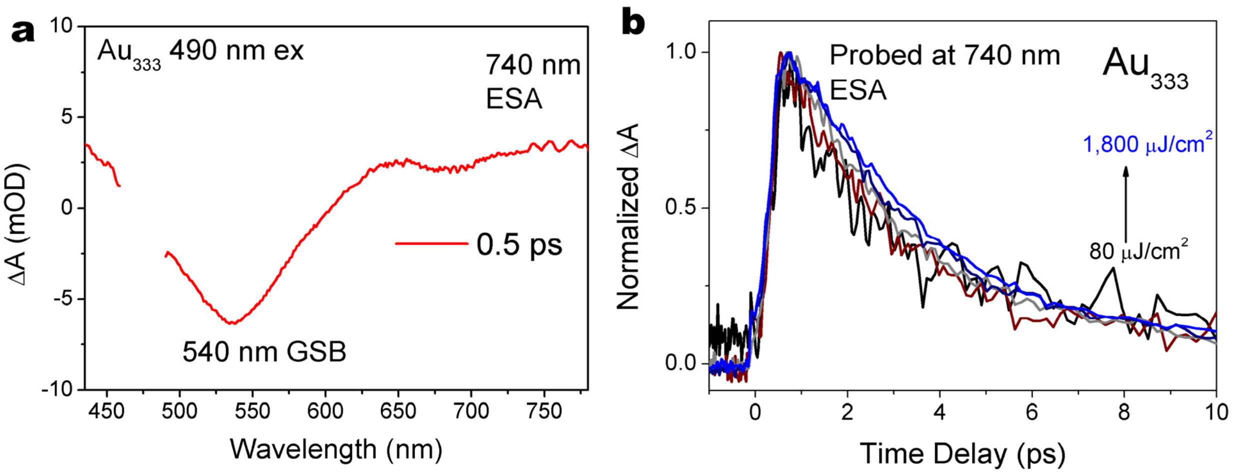

Supplementary Figure 9. Power dependent electron dynamics of Au_{333}. (a) Transient absorption spectra at 0.5 ps, residual scattering of the pump beam around 480 nm was removed. (b) Kinetic traces of ESA around 740 nm as a function of pump fluence, which exhibits weak power dependence.

Supplementary Note

Supplementary Note 1. Detailed preparation method and characterization of gold nanoparticles.

Sample Preparation and Characterization. All these six nanoclusters were synthesized based on the size-focusing methodology.[1] Briefly, in the first step, a mixture of $Au_x(SR)_y$ nanoclusters was synthesized by reducing Au(I)SR polymers with $NaBH_4$. By adjusting the reaction conditions, the $Au_x(SR)_y$ mixture was controlled to be in a proper size range. In the following step, harsh conditions (i.e. large excess of thiol and high temperature) were applied to select the stable magic sizes in the size range. The Au_{25}, Au_{38}, Au_{144}, Au_{333}, $Au_{\sim520}$ and $Au_{\sim940}$ were all protected by the same ligand (i.e., phenylethanethiolate). The former four sizes were synthesized based on the previously developed protocols,[2-5] and a modification of the reported protocol[5] by reducing the thiol-to-gold salt ratio below 2:1 led to the next $Au_{\sim940}$ magic size.

Supplementary Figure 1shows the matrix-assisted laser desorption/ionization mass spectroscopy (MALDI-MS) of the later four nanoclusters. The precise molecular weight (MW) determination requires electron-spray ionization mass spectroscopy (ESI-MS);[5] see Supplementary Figure 1 for Au_{25}, Au_{38}, Au_{144} and Au_{333}. But as the size of gold nanoclusters increases further, determination of the precise MW and hence formula becomes a daunting task, which is largely due to the low ionization efficiency of giant nanoclusters in ESI-MS and also many possible combinations of (n, m) in $Au_n(SR)_m$ for the same MW. Here, we use a facile method to estimate the formula of giant nanoclusters, based on laser-intensity-dependent MALDI-MS. As shown in Supplementary Figure 1 for Au_{25}, Au_{38}, Au_{144} and Au_{333}. But as the size of gold nanoclusters increases further, determina, the intact molecular weight of $Au_n(SR)_m$ can be estimated from the onset of ionization peak measured in low laser intensity (denoted as MW_{LI}). When increasing the laser intensity, fragmentation occurs due to the break of S-R bond in thiolate, and the peak in high laser intensity (MW_{HI}) is approximately correlated to Au_nS_m. Thus, the m value can be calculated by $(MW_{LI} - MW_{HI})/MW_R$, where MW_R is molecular weight of the R group in thiolate. Then, the n value can be determined.

Using this method, the 34k, 74k, 115k, and 200k species (k=1000) shown in Figure 1a have MW_{LI} of 36.5k, 75.6k, 116.6k and 207.5k, and MW_{HI} of 28.9k, 66.0k, 105.7k, and 190.0k, and thus the formulas are calculated to be $Au_{\sim135}(SR)_{\sim72}$, $Au_{\sim317}(SR)_{\sim95}$, $Au_{\sim519}(SR)_{\sim104}$, and $Au_{\sim939}(SR)_{\sim164}$, respectively. The main uncertainty in this method is from MW_{LI}, since the fragmentation would lead to further cleavage of Au-S bonds, making the value of MW_{LI} in between Au_n and Au_nS_m. Thus, the determined m value is often lager than the theoretical one, while the n value being smaller. This is reflected through the comparison with precisely determined $Au_{144}(SR)_{60}$ and $Au_{333}(SR)_{79}$ formulas for the 34k and 74k nanoclusters by high precision ESI-MS.[4,5]

Supplementary Note 2. Data analysis on the electron dynamics of gold nanoparticles.

The ΔA as a function of time delay for plasmonic gold nanoparticles were fitted by convoluting the theoretical equation with the instrument response function:[6,7]

$$\Delta A(t)=\int_{-\infty}^{+\infty}\{H(\tau)[A(1-e^{-\tau/\tau_{\text{e-e}}})e^{-\tau/\tau_{\text{e-ph}}}+B(1-e^{-\tau/\tau_{\text{e-ph}}})]\}e^{-(t-\tau)^2/\tau_0^2}\mathrm{d}\tau$$

where $H(\tau)$ is the Heaviside function, $\tau_{\text{e-e}}$ and $\tau_{\text{e-ph}}$ are decay times due to the electron-electron scattering and electron phonon coupling, respectively. τ_0 is determined by the cross correlation of the pump and probe pulses. This equation considers electronic response ($(1-e^{-\tau/\tau_{\text{e-e}}})e^{-\tau/\tau_{\text{e-ph}}}$) which rises due to electron-electron scattering and decay due to e-p coupling, as well as other effects $(1-e^{-\tau/\tau_{\text{e-ph}}})$ due to heating of the lattice. The fitting results were shown in Supplementary Figure 4, $\tau_{\text{e-ph}}$ were used as the electron phonon coupling time constants in the main text.

Supplementary Note 3. Kinetic traces of Au_{144} at different wavelengths and with different pump wavelengths.

Previous work has reported that Au_{144} exhibit pump power dependent electron dynamics and thus Au_{144} is the onset of metallic behavior.[8,9] However, we found that Au_{144} shows power independent electron dynamics by carrying out TA experiments at different pump and probe wavelengths. Supplementary Figure 5 shows the kinetic traces around 460 nm and 525 nm with 490 nm pump. It can be seen that GSB around 525 nm is overlapped with ESA and became positive after 5 ps. Therefore, we chose GSB around 460 nm which is less overlapped with ESA to monitor the power dependence.

Supplementary Figure 6 shows the kinetic traces around 525 nm and 460 nm as a function of pump fluence. It can be seen that GSB around 460 became positive at time delay after 5 ps. We have reproduced Yi *et al.* results by fitting the kinetic traces at 525 nm using a two exponential function convoluted with instrument response function, which somehow shows power dependence. However, when we monitored the kinetic traces at 460 nm and other wavelengths, the decay traces at all pump fluences give the same decay time constants. Supplementary Figure 8 shows the transient absorption spectra of Au_{144} with 370 nm and 700 nm pump. It shows clearly that under both 370 nm and 700 nm excitations, the electron dynamics of Au_{144} is independent of the pump power. These results demonstrate that Au_{144} exhibit non-metallic behavior.

Supplementary Note 4. Time evolution of transient absorption spectra and kinetic traces of $Au_{25}(SR)_{18}$ and $Au_{38}(SR)_{24}$.

Supplementary Figure 8 shows the transient spectral features and kinetic traces at selected wavelengths for $Au_{25}(SR)_{18}$ and $Au_{38}(SR)_{24}$ nanoclusters. Unlike plasmonic gold nanoparticles, these small gold nanoclusters exhibit multi excited state absorptions (ESA) overlapped with ground state bleaching (GSB) signals all over the visible region (Supplementary Figure 8 a and c). Their excited state lifetime is relatively longer than that of larger gold nanoparticles, the GSB signal for both Au_{25} and Au_{38} clusters does not decay to zero at 3 ns. With discrete UV-vis absorption, large bandgap and longer excited state lifetime, both Au_{25} and Au_{38} nanoclusters exhibit typical molecular behavior.

Supplementary References

1. Jin, R., *et al.* Size Focusing: A Methodology for Synthesizing Atomically Precise Gold Nanoclusters. *J. Phys. Chem. Lett.* **1**, 2903-2910 (2010).
2. Wu, Z., Suhan, J. & Jin, R. One-Pot Synthesis of Atomically Monodisperse, Thiol-Functionalized Au_{25} Nanoclusters. *J. Mater. Chem.* **19**, 622-626 (2009).
3. Qian, H., Zhu, Y. & Jin, R. Size-Focusing Synthesis, Optical and Electrochemical Properties of Monodisperse $Au_{38}(SC_2H_4Ph)_{24}$ Nanoclusters. *ACS Nano* **3**, 3795-3803 (2009).
4. Qian, H. & Jin, R. Controlling Nanoparticles with Atomic Precision: The Case of $Au_{144}(SCH_2CH_2Ph)_{60}$. *Nano Lett.* **9**, 4083-4087 (2009).
5. Qian, H., Zhu, Y. & Jin, R. Atomically precise gold nanocrystal molecules with surface plasmon resonance. *Proc. Natl. Acad. Sci. U.S.A.* **109**, 696-700 (2012).
6. Voisin, C., *et al.* Ultrafast electron-electron scattering and energy exchanges in noble-metal nanoparticles. *Phys. Rev. B* **69**, 195416 (2004).
7. Tang, Y. & Ouyang, M. Tailoring properties and functionalities of metal nanoparticles through crystallinity engineering. *Nat Mater* **6**, 754-759 (2007).
8. Yi, C., Tofanelli, M.A., Ackerson, C.J. & Knappenberger, K.L. Optical Properties and Electronic Energy Relaxation of Metallic $Au_{144}(SR)_{60}$ Nanoclusters. *J. Am. Chem. Soc.* **135**, 18222-18228 (2013).
9. Mustalahti, S., *et al.* Ultrafast Electronic Relaxation and Vibrational Cooling Dynamics of $Au_{144}(SC_2H_4Ph)_{60}$ Nanocluster Probed by Transient Mid-IR Spectroscopy. *J. Phys. Chem. C* **118**, 18233-18239 (2014).
